# Cytotoxic Effects of Newly Synthesized Heterocyclic Candidates Containing Nicotinonitrile and Pyrazole Moieties on Hepatocellular and Cervical Carcinomas

**DOI:** 10.3390/molecules24101965

**Published:** 2019-05-22

**Authors:** Amira A. El-Sayed, Abd El-Galil E. Amr, Ahmed K. EL-Ziaty, Elsayed A. Elsayed

**Affiliations:** 1 Laboratory of Synthetic Organic Chemistry, Chemistry Department, Faculty of Science, Ain Shams University, Abbassia, Cairo 11566, Egypt; ahm512@gmail.com; 2 Pharmaceutical Chemistry Department, Drug Exploration & Development Chair (DEDC), College of Pharmacy, King Saud University, Riyadh 11451, Saudi Arabi; 3 Applied Organic Chemistry Department, National Research Center, Cairo, Dokki 12622, Egypt; 4 Zoology Department, Bioproducts Research Chair, Faculty of Science, King Saud University, Riyadh 11451, Saudi Arabiap; eaelsayed@ksu.edu.sa; 5 Chemistry of Natural and Microbial Products Department, National Research Centre, Dokki 12622, Cairo, Egypt

**Keywords:** cyanopyridone, substituted pyridine, pyridotriazine, pyrazolopyridine, thioxotriazopyridine, anticancer activity, HepG2, HeLa

## Abstract

In this study, a series of newly synthesized substituted pyridine **9, 11**–**18**, naphthpyridine derivative **10** and substituted pyrazolopyridines **19**–**23** by using cycnopyridone **8** as a starting material. Some of the synthesized candidates are evaluated as anticancer agents against different cancer cell lines. In vitro cytotoxic activities against hepatocellular and cervical carcinoma cell lines were evaluated using standard MTT assay. Different synthesized compounds exhibited potential in vitro cytotoxic activities against both HepG2 and HeLa cell lines. Furthermore, compared to standard positive control drugs, compounds **13** and **19** showed the most potent cytotoxic effect with IC_50_ values of 8.78 ± 0.7, 5.16 ± 0.4 μg/mL, and 15.32 ± 1.2 and 4.26 ± 0.3 μg/mL for HepG2 and HeLa cells, respectively.

## 1. Introduction

**Figure 1:**
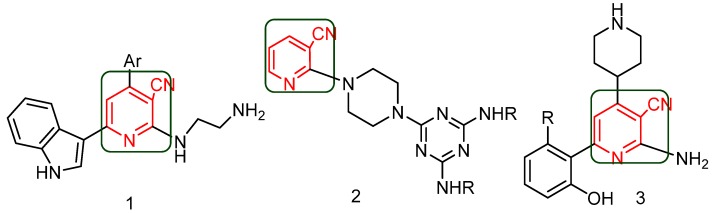
Nicotinonitriles with anti-proliferative, anti-Alzheimer’s anti-inflammatory activities.

Multicomponent reactions (MCR) “in which three or more starting materials react to form a product” play a significant role in the synthesis of heterocyclic compounds with pharmaceutical and chemical importance [1]. Several nicotinonitriles have been constructed via (MCR) and showed antitumor [2], antimicrobial [3], and antioxidant [4] activities. Also nicotinonitriles have been utilized as a scaffold for the synthesis of heterocyclic compounds containing a pyridine moiety with antimicrobial and antiviral activities [5]. A series of nicotinonitriles **1**–**3** (Figure 1) and have been synthesized and anti-proliferative [6], anti-Alzheimer’s [7], and anti-inflammatory [8] activities.

**Figure 2:**
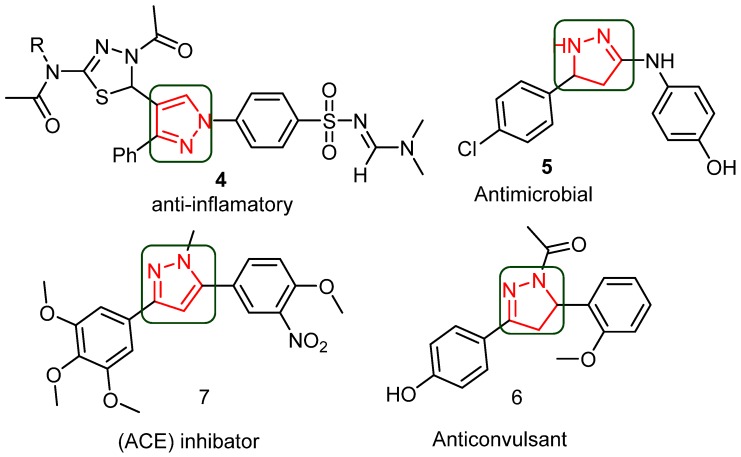
Pyrazoles as anti-inflammatory antimicrobial and anticonvulsant activities.

The pyrazole moiety is both pharmacologically and medicinally significant [9]. A series of pyrazoles **4**–**7** (Figure 2) has been reported as anti-inflammatory activity by Bekhit et al. [10], they observed that the synthesized pyrazoles showed more anti-inflammatory activity than the standard indomethacin [11]. Trisubstituted pyrazoles have been constructed by Christodoulou et al. (2010) [11] and evaluated as anti-angiogenic agents; these derivatives showed a potent anti-angiogenic efficacy and moreover inhibited the growth of Mammary gland breast cancer (MCF-7) and cervical carcinoma (Hela) [12]. Recently novel derivatives of pyrazoles **5**,**6** have been prepared as antimicrobial [13] and anticonvulsant [14] agents. The pyrazole **7** has been prepared by Bonesi et al. (2010) [15] and showed effective Angiotensin -1-Converting Enzyme (ACE) inhibitor activity [15].

Based on the previous facts about the importance of pyrazoles and nicotinonitriles in medicinal chemistry, we have herein synthesized of some novel heterocyclic candidates containing nicotinonitrile and pyrazole moieties and tested their anticancer activity. 

## 2. Results

### 2.1. Chemistry

**Scheme 1:**
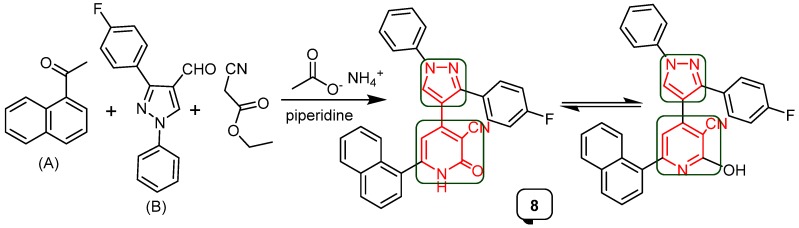
Synthesis of compound **8** as starting material.

The nicotinonitriles were obtained by two different ways, from the reaction of chalcone with ethylcyanoacetate, ammonium acetate and drops of piperidine as a base and from one pot four components reaction of methylketone, aldehyde, ethylcyanoacetate, ammonium acetate and drops of piperidine as a base [15]. In prolongation of our work in the synthesis of heterocyclic compounds and evaluation of their medicinal importance [16,17,18,19,20,21,22,23,24,25,26,27] and based on the literature survey about the pharmacological and medicinal importance of pyrazoles and nicotinonitriles, we have devoted our efforts to design and synthesize novel heterocyclic compounds containing pyrazol and nicotine-nitrile moieties, 4-(3-(4-fluorophenyl)-1-phenyl-1*H*-pyrazol-4-yl)-2-hydroxy-6-(naphthalen-1-yl)-nicotinenitrile **8** has been obtained by reacting of 1-acetylnaphthalene (**A**), 3-(4-fluorophenyl)-1-phenyl-1*H*-pyrazole-4-carbaldehyde (**B**), ethyl 2-cyanoacetate, ammonium acetate and piperidine (Scheme 1).

The structure of the nicotinonitrile **8** has been confirmed from its spectral data. IR spectrum showing absorption frequencies at ν 3159 cm^−1^, 2220 cm^−1^ and ν 1647 cm^−1^ for OH, C≡N and C=N groups, respectively. Also, ^1^H-NMR spectrum of the assigned compound displayed signals at δ 12.89 ppm (disappeared with D_2_O) corresponding to acidic OH. A compelling evidence for the structure of **8** was provided by ^13^C-NMR spectrum that showed a singlet signal at δ 149.8, 139.3 and 139.3 ppm for C-OH, C=N and C≡N groups respectively. Mass spectra of **8** showed [M^+^] at *m*/*z* (%) 482 (22). Treatment of **8** with ethylchloroacetate afforded compound **9**, which was hydrazinolysis with NH_2_NH_2_ to give the corresponding cyclized product **10**. 

**Scheme 2:**
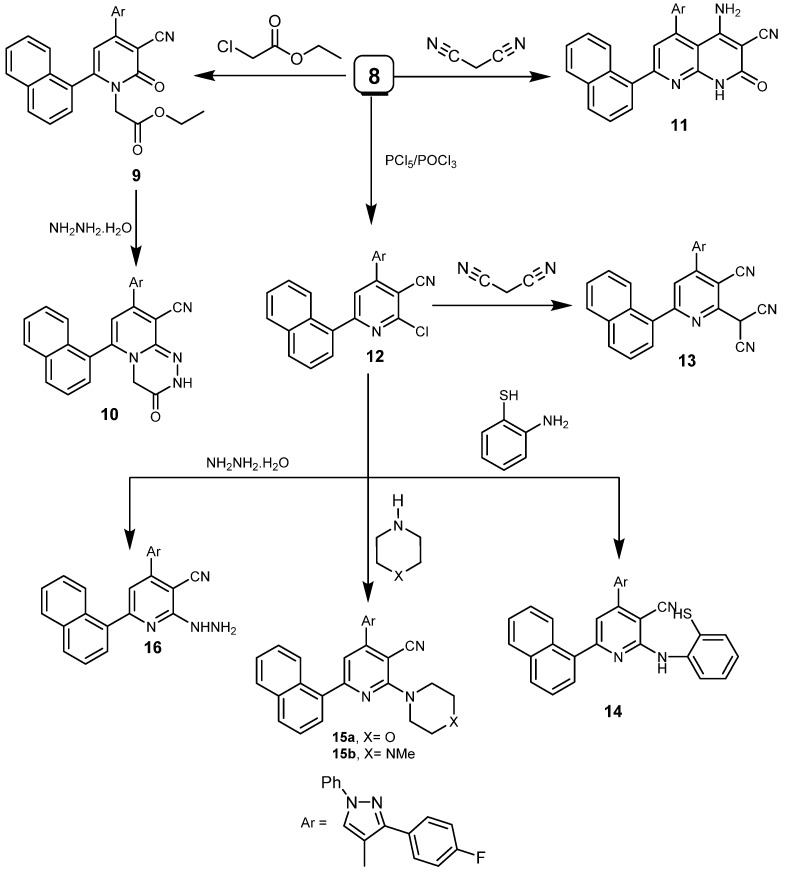
Synthetic route for compounds **9**–**16**.

**Figure 3:**
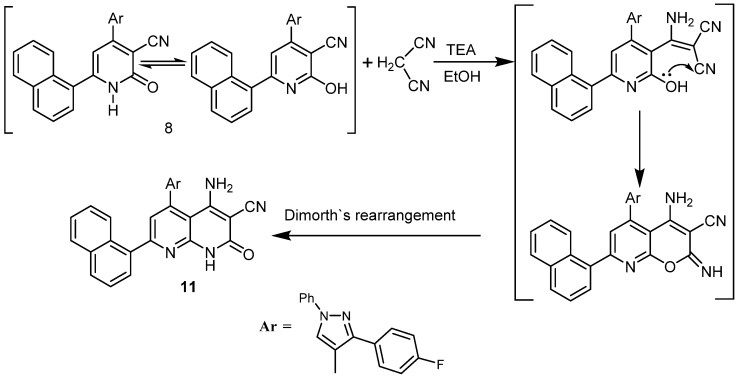
The mechanism formation route of compound **11**.

Remediation of the nicotinonitrile derivative **8** with malononitrile in the presence of few drops of piperidine afforded 1,8-naphthyridine-3-carbonitrile derivative **11**. Chlorination of **8** by a mixture of (POCl_3_/PCl_5_) afforded 2-chloronicotinonitrile derivative **12**, which was reacted with malono nitrile as a carbon nucleophile gave the nicotinonitrile derivative **13**. Reaction of **12** with primary and secondary amines, namely, *o*-aminothiophenol, morpholine, 1-methylpiperazine and hydrazine hydrate gave novel nicotinonitriles **14**, **15a**, **b** and **16** (Scheme 2). The mechanism formation route of compound **11** has been shown in Figure 3.

**Scheme 3:**
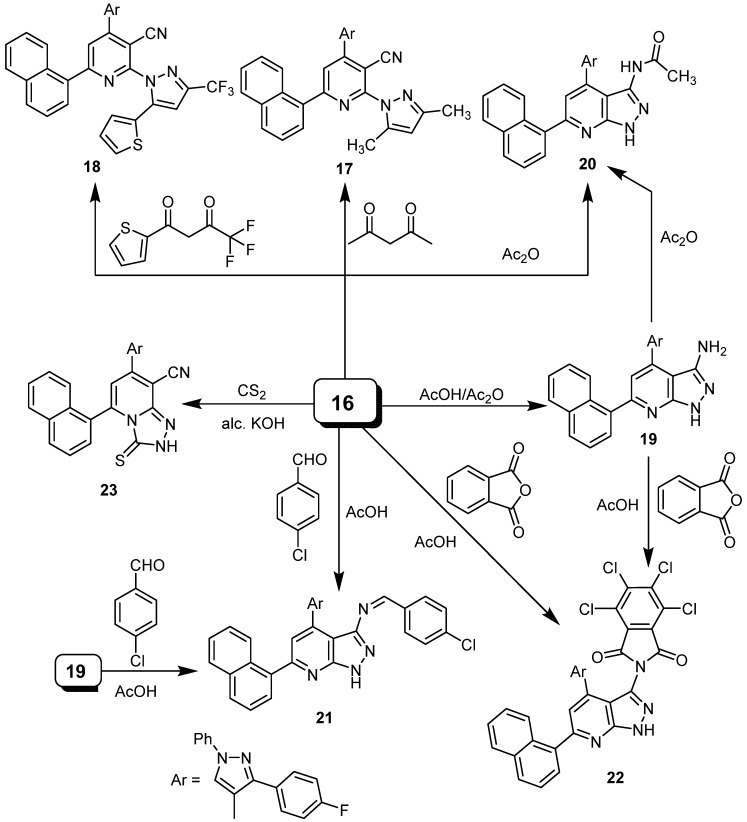
Synthetic route for compounds **17**–**23**.

Compound **16** was utilized as a building block for novel nicotinonitriles containing two pyrazole moieties. 2-Pyrazolyl nicotinonitrile derivatives **17** and **18** were prepared by treatment of **16** with acetyl acetone and 4,4,4,-trifluoro-1-(thiophen-2-yl)butane-1,3-dione, respectively. Treatment of **16** with acetic anhydride and acetic acid afforded pyrazolopyridine derivative **19**. The derivative **16** was treated with acetic anhydride to afford the *N*-acetyl pyrazolopyridine as a sole product **20**. The structure of compound **20** was confirmed chemically by acetylation of the amino pyrazopyridine **19** (Scheme 3).

Treatment of **16** with 4-chlorobenzaldehyde and/or tetrachlorophthalic anhydride in the presence of acetic acid afforded the cyclized **19** followed by condensation to give the Schiff’s base **21** and tetra chloroisoindoline **22**, respectively. The structures of **21** and **22** were confirmed chemically by condensation of compound **19** with 4-chlorobenzaldehyde and/or tetrachlorophthalic anhydride to provide compounds **21** and **22**, respectively. Treatment of hydrazinyl derivative **16** with CS_2_ in the presence of alcoholic KOH provided thioxotriazolo pyridine derivative **23** (Scheme 3).

### 2.2. Cytotoxic Activity

The newly synthesized compounds were screened for their anticancer potentials against hepatocellular carcinoma HepG2 and cervical carcinoma HeLa. The cytotoxicity of the compounds was determined using MTT assay and DOX as a positive control [28,29,30,31]. 

**Figure 4:**
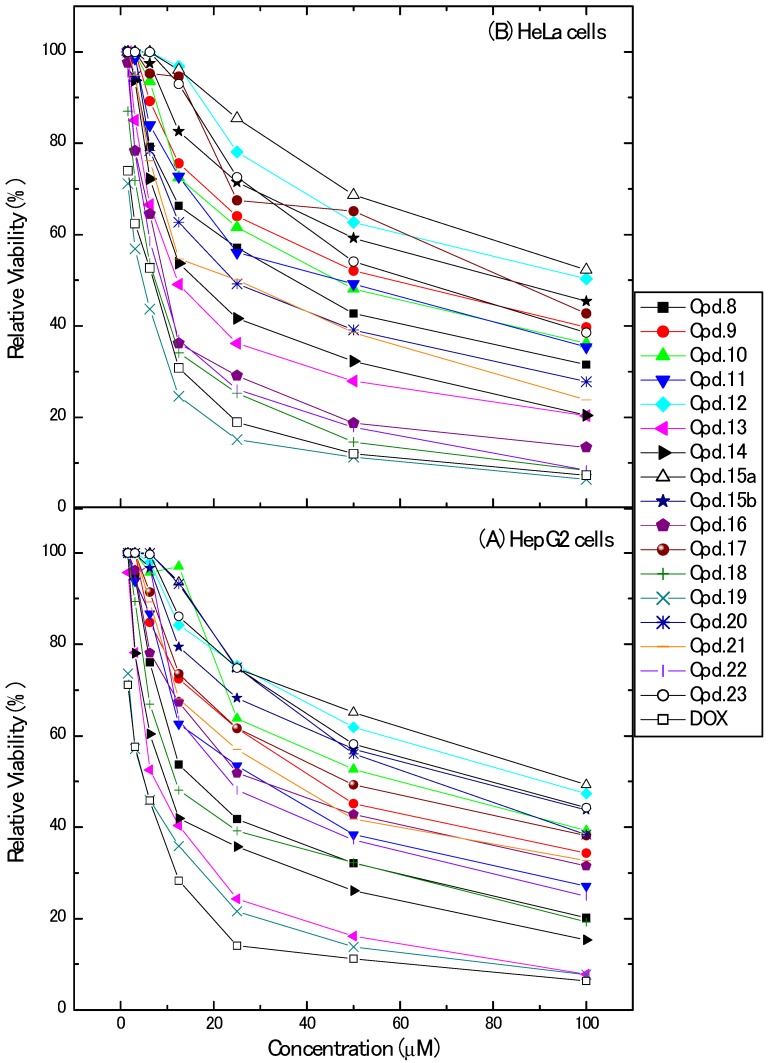
Relative viabilities of HepG2 and HeLa cells as affected by different synthesized compounds.

The cytotoxic activities of the novel synthesized compounds **8**–**23** were estimated and the obtained results are presented in Figure 4. In general, it can be seen that all synthesized compounds exhibited cytotoxic activities against both tested cancer cell lines. Moreover, it can be seen that both cells reacted in a dose-dependent manner toward the applied concentrations. Additionally, both tested cell lines varied in their response toward different synthesized compounds. Furthermore, based on the IC_50_ values (Table 1) obtained for the tested compounds, it can be seen that cytotoxic activities ranged from very strong to non-cytotoxic. Compounds **13** and **19** exhibited the most potent cytotoxic effect (very strong activity) with IC_50_ 8.78 ± 0.7, 5.16 ± 0.4 μg/mL, and 15.32 ± 1.2 and 4.26 ± 0.3 μg/mL for HepG2 and HeLa cells, respectively. Furthermore, it can be noticed that **Cpd. 19** exhibited more or less stronger activity similar to DOX towards HepG2 cells, (IC_50_ 5.16 ± 0.4 and 4.50 ± 0.2 μg/mL, respectively). On the other hand, it was stronger by about 23.5% than DOX against HeLa cells (4.50 ± 0.2 and 5.57 ± 0.4 μg/mL, respectively). Additionally, **Cpd. 18** showed very strong activity towards HeLa cells with IC_50_value of 7.67 ± 0.6 μg/mL, while it exhibited strong activity towards HepG2 cells (IC_50_ 16.70 ± 1.3 μg/mL). Moreover, **Cpd. 14** showed strong cytotoxic activities towards both tested cell lines (IC_50_values 12.20 ± 1.0 and 19.44 ± 1.4 μg/mL for HepG2 and HeLa cells, respectively). Meanwhile, **Cpds. 16** and **22** showed moderate and strong activities towards both cell lines. **Cpd. 16** showed IC_50_value of 33.45 ± 2.3 and 10.37 ± 0.9 μg/mL against HepG2 and HeLa cells, respectively. Also, **Cpd. 22** showed IC_50_ of 26.64 ± 1.9 and 9.33 ± 0.8 μg/mL for HepG2 and HeLa cells, respectively. On the other hand, **Cpd. 17** showed strong activity towards HepG2 cells (IC_50_ 20.00 ± 1.7 μg/mL) and moderate activity towards HeLa cells (IC_50_ 35.58 ± 2.6 μg/mL). Finally, **Cpds. 9**, **10**, **11**, **12**, **15a**, **b**, **17**, **20**, **21** and **23** showed activities ranging from moderate to non-cytotoxic, with IC_50_ values ranging from 24.83 ± 1.8 to >100 μg/mL.

## 3. Discussion

During current work, multi-component reaction strategy was used to synthesize of compound **8**, which was used as a building block for preparing **16** new derivatives. The cytotoxic potential of the new prepared compounds has been evaluated against HepG2 and HeLa cells. Results obtained showed potential cytotoxic activities against both cell lines. Compounds **13** and **19** showed the most cytotoxic effects (IC_50_ 8.78 ± 0.7 and 5.16 ± 0.4 μg/mL, for HepG2 cells, and 15.32 ± 1.2 and 4.26 ± 0.3 μg/mL for HeLa cells, respectively). Also, results showed that both tested cell lines varied in their response toward different synthesized compounds. This can be attributed to the inherent differences in both cell lines in terms of membrane structure and organization, hence different cell lines react differently towards different compounds [32,33,34,35].

Different activities of the prepared compounds may be attributed to the structure–activity relationship of these compounds. It can be seen that conversion of **Cpd. 12** to **13**, **14** and **16**, **18**, **19** and **22** altered the cytotoxicity from weak to moderate and strong activity towards two cell lines. This explained due to the introduction of two more nitrile groups, which significantly increased the activity. Compound **14** exhibited very strong activity due to the entity of the SH and NH groups, which may be added to any unsaturated group in DNA (thia or aza Michael addition) or the formation of hydrogen bonds with either one of the nucleo-bases of the DNA, thus causing DNA damage. Furthermore, the cytotoxicity of **Cpd. 16** may be due to the intermolecular hydrogen bonding of NH and NH_2_ groups with DNA moieties. Additionally, conversion of **Cpd. 16** to **18**, **19** and **22** increased their cytotoxic activities against both cell lines. Introducing thiophene ring increases the cytotoxic effect of **Cpd. 18** beside the effect of the pyrazole ring and the trifluoromethyl group. Additionally, introducing pyrazole ring bearing NH_2_ group to **Cpd. 16** increases the cytotoxic effect of **Cpd. 19** to very strong effect against both cell lines. The introduction of chloroiso- indoline-1,3-dione increases the cytotoxic effects of **Cpd. 22**. The chloro- group, with more electron withdrawing properties, may be the crucial for tumor cell inhibition beside the effect of the isoindoline-1,3-dioneas moderate cytokine inhibitor in cancer cells.

## 4. Materials and Methods 

### 4.1. Chemistry

“Melting points reported are inaccurate. IR spectra were registered on Shimadzu FT-IR 8300 E (Shimadzu Corporation, Kyoto, Japan) spectrophotometer using the (KBr) disk technique. ^1^H-NMR spectra were determined on a Varian Spectrophotometer at 400 MHz using (TMS) as an internal reference and DMSO-d_6_ as solvent using (TMS) as internal standard. All chemical shifts (δ) are uttered in ppm. The mass spectra were determined using (MP) model MS-5988 and Shimadzu single focusing mass spectrophotometer (70 eV). Elemental analysis was investigated by Elemental analyzer Vario EL III”.

#### 4.1.1. Synthesis of 4-(3-(4-fluorophenyl)-1-phenyl-1*H*-pyrazol-4-yl)-2-hydroxy-6-(naphthalen-1-yl)-nicotinenitrile (**8**)

A mixture of 1-acetyl naphthalene (**A**) (1.7 g, 0.01 mol), ethyl cyanoacetate (1.3 g, 0.01 mol), aldehyde (**B**) (3.6 g, 0.01 mol), ammonium acetate (5.40 g, 0.07 mol) and three drops of piperidine in ethanol (20 mL) was heated under reflux for 3 h. The obtained precipitate was filtered off, washed with cold water, dried and crystallized from ethanol/dioxane to give compound **8**. Yield 75%, yellow powder, m.p. > 300 °C; IR (KBr): ν (cm^−1^) 3159 (OH), 2220 (C≡N), 1647 (C=N); ^1^H-NMR (DMSO-d_6_): δ (ppm) 12.89 (s, 1H, OH, disappeared by D_2_O), 9.80 (s, 1H, pyrazole-H), 8.39–7.78 (m, 7H, Ar-H for naphthalene), 7.75–7.37 (m, 10H, Ar-H). ^13^C NMR (DMSO-d_6_): δ (ppm) 149.8 (C-OH), 139.3 (C=N), 119.3 (C≡N), 139.4, 134.3, 133.8, 133.5, 131.6, 131.2, 131.0, 130.9, 130.4, 130.3, 130.2, 129.9, 129.4, 129.3, 129.2, 129.1, 128.9, 128.2, 127.8, 127.6, 127.0, 125.6, 125.1, 117.4, 114.8 (Ar-CH), 40.6, 39.9 (aliph-C); MS *m*/*z* (ESI): 482 [M^+^] (22), 465 (21), 440 (12), 237 (100), 204; Anal. Calcd. for C_31_H_19_FN_4_O (482.50): C, 77.17; H, 3.97; N, 11.61. Found C, 76.98; H, 3.78; N, 11.52%.

#### 4.1.2. Synthesis of ethyl 2-(3-cyano-4-(3-(4-fluorophenyl)-1-phenyl-1*H*-pyrazol-4-yl)-6-(naphthalene-1-yl)-2-oxopyridin-1(2*H*)-yl)acetate (**9**)

A mixture of **8** (4.84 g, 0.01 mol), ethylchloroacetate (1.22 g, 0.01 mol) and K_2_CO_3_ (2.2 g, 0.015 mol) in (CH_3_)_2_O (40 mL) was heated under reflux for 24 h, concentrated and poured on water; the obtained precipitate was collected by filteration off, dried and crystallized from EtOH/dioxane to give **9**. Yield 74%, m.p. 158–160 °C; IR (KBr): ν (cm^−1^) 2204 (C≡N), 1751 (C=O ester), 1651 (C=O pyridine); ^1^H-NMR (DMSO-d_6_): δ (ppm) 9.15 (s, 1H, pyrazole-5H), 8.10–7.49 (m, 7H, Ar-H for naphthalene), 7.48–7.33 (m, 10H, Ar-H), 4.16 (q, 2H, -CH_2_ ester), 3.40 (s, 2H, -CH_2_), 1.20 (t, 3H, -CH_3_, ester); MS *m*/*z* (ESI): 568 [M^+^] (2.5), 495 (65), 237 (80), 127 (100); Anal. Calcd. for C_35_H_25_FN_4_O_3_ (568.60): C, 73.93; H, 4.43, N, 9.85. Found C, 73.80; H, 4.21; N, 9.64%.

#### 4.1.3. Synthesis of 8-(3-(4-fluorophenyl)-1-phenyl-1*H*-pyrazol-4-yl)-6-(naphthalen-1-yl)-3-oxo-3,4-dihydro-2*H*-pyrido[2,1-c][1,2,4]triazine-9-carbonitrile (**10**)

A mixture of **9** (5.7 g, 0.01 mol), NH_2_NH_2_ηH_2_O (2 mL, 0.04 mol) and EtOH (20 mL) was heated under reflux for 3 h. The outward appearance solid was filtered off, dried and crystallized from EtOH/dioxane to give **10**. Yield 71%, yellow powder, m.p. > 300 °C; IR (KBr): ν (cm^−1^) 3209 (NH), 2218 (C≡N), 1647 (C=O); ^1^H-NMR (DMSO-d_6_): δ (ppm) 12.38 (s, 1H, NH, disappeared in D_2_O), 9.13 (s, 1H, pyrazole-5H), 8.87–7.65 (m, 7H, Ar-H for naphthalene), 7.63–6.85 (m, 10H, Ar-H), 6.10 (s, 2H, CH_2_). ^13^C-NMR (DMSO-d_6_): δ (ppm) 165.8 (C=O), 139.7 (C=N), 136.1 (C=N), 133.8, 133.4, 131.7, 130.9, 130.8, 130.7, 130.6, 130.3, 130.2, 130.1, 129.9, 129.7, 129.2, 129.1, 128.8, 128.5, 128.1, 127.3, 126.8, 125.8, 125.6, 119.2, 119.1, 118.9, 118.5, 117.6 (Ar-CH), 119.3 (C≡N), 40.5, 39.9 (2CH), 17.6 (CH_2_); MS *m*/*z* (ESI): 519 [M^+^ − OH] (82), 393 (64), 284 (100), 237 (68), 127 (56); Anal. Calcd. for C_33_H_21_FN_6_O (536.50): C, 73.87; H, 3.94; N, 15.66. Found C, 73.68; H, 3.24; N, 15.06%.

#### 4.1.4. Synthesis of 5-(3-(4-fluorophenyl)-1-phenyl-1*H*-pyrazol-4-yl)-7-(naphthalen-1-yl)-2-oxo-1,2-dihydro-1,8-naphthyridine-3-carbonitrile (**11**)

Refluxing of compound **8** (4.84 g, 0.01 mol) with malononitrile (0.015 mol) in ethanol (20 mL) in the presence of drops of TEA for 5 h, then cooled, poured on ice/water, neutralized with drops of conc. HCl. The obtained solid was collected by filtration, crystallized from EtOH/dioxane to afford **11**. Yield 71%, pale brown powder, m.p. > 300 °C; IR (KBr): ν (cm^−1^) 3386, 3273 (NH_2_), 3158 (NH), 2218 (C≡N), 1646 (C=O), ^1^H-NMR (DMSO-d_6_): δ (ppm) 12.89 (s, 1H, NH, disappeared by D_2_O), 9.08 (s, 1H, pyrazole-5H), 8.07–7.61 (m, 7H, Ar-H for naphthalene), 7.60–7.37 (m, 10H, Ar-H), 6.22 (s, 2H, NH_2_, disappeared in D_2_O). ^13^C-NMR (DMSO-d_6_): δ (ppm) 149.9 (C=O), 139.3 (C=N), 133.8, 133.5, 131.2, 131.1, 131.00 (2), 130.9, 130.4, 130.3 (2), 130.2, 129.9 (2), 129.4, 129.1, 128.9 (2), 128.2, 127.8(2), 127.6, 127.1 (2), 125.6, 125.2 (2), 117.4, 116.8, 110.0 (Ar-CH), 119.3 (C≡N), 40.6, 39.9 (2CH); MS *m*/*z* (ESI): 532 [M^+^ − NH_3_] (82), 516 (76), 440 (28), 310 (20), 237 (100); Anal. Calcd. for C_34_H_21_FN_6_O (548.50): C, 74.44; H, 3.89; N, 15.32. Found C, 74.24; H, 3.25; N, 14.98%.

#### 4.1.5. Synthesis of 2-chloro-4-(3-(4-fluorophenyl)-1-phenyl-1*H*-pyrazol-4-yl)-6-(naphthalen-1-yl)-nicotinenitrile (**12**)

A mixture of **8** (4.82 g, 0.01 mol), PCl_5_ (3 g, 0.03 mol) and POCl_3_ (5 mL, 0.03 mol) was heated under reflux for 8 h, then it was poured on crushed ice. The formed solid was filtered off, dried and crystallized from EtOH/dioxane to give **12**. Yield 61%, yellow powder, m.p. 164–166 °C; IR (KBr): ν (cm^−1^) 2227 (C≡N), 1628 (C=N); ^1^H-NMR (DMSO-d_6_): δ (ppm) 9.16 (s, 1H, pyrazole-5H), 8.35–7.63 (m, 7H, Ar-H for naphthalene), 7.61–7.39 (m, 10H, Ar-H). ^13^C-NMR (DMSO-d_6_): δ (ppm) 152.7, 150.0, 148.4, 139.2 (C=N), 135.3(C=N), 133.8, 131.5, 131.0, 130.4, 130.3, 130.2, 129.8, 129.5, 129.2 (2), 129.1, 127.9, 127.6, 126.9, 125.8 (2), 125.4, 125.1, 119.3 (C≡N), 116.6, 115.5, 107.8 (Ar-CH), 40.6, 39.9 (2CH); MS *m*/*z* (ESI): 503 [M^+^ + 2] (6), 501 [M^+^] (50), 465 (100), 237 (82); Anal. Calcd. for C_31_H_18_ClFN_4_ (500.90): C, 74.32; H, 3.62; N, 11.84. Found C, 74.12; H, 3.26; N, 11.42%.

#### 4.1.6. Synthesis of 2-[4-(3-(4-fluorophenyl)-1-phenyl-1*H*-pyrazol-4-yl)-6-(naphthalen-1-yl)-3-cyano-pyridinyl]malononitrile (**13**)

To a solution of **12** (5.0 g, 0.01 mol) in EtOH (20 mL), malononitrile (0.01 mol) and TEA (1 mL) were added. The reaction mixture was heated under for 3 h. After cooling, it was poured on water and neutralized with diluted HCl. The obtained solid was separated by filtration, washed with water, dried and crystallized from EtOH/dioxane to yield **13**. Yield 76%, pale brown powder, m.p. 194–196 °C; IR (KBr): ν (cm^−1^) 2203 (C≡N), ^1^H-NMR (DMSO-d_6_): δ (ppm) 9.15 (s, 1H, pyrazole-5H), 8.11–7.66 (m, 7H, Ar-H for naphthalene), 7.65–7.36 (m, 10H, Ar-H), 7.07 (s, 1H, CH of CH(CN)_2_), MS *m*/*z* (ESI): 530 [M^+^] (12), 440 (100), 237 (76), 204 (31); Anal. Calcd. for C_34_H_19_FN_6_ (530.50): C, 76.97; H, 3.61; N, 15.84. Found C, 76.78; H, 3.42; N, 15.24%.

#### 4.1.7. Synthesis of **14** and **15a**,**b**

A mixture of 2-chloronicotinonitrile **12** (5.0 g, 0.01 mol) and the appropriate amine, namely, o-aminothiophenol, morpholine or 2-methylpiperidine (0.01 mol) in EtOH (20 mL) was heated under reflux for 3 h, then it was poured on cold water, filtered off and crystallized from EtOH/dioxane to afford **14** and **15a,b**, respectively.

4-(3-(4-Fluorophenyl)-1-phenyl-1*H*-pyrazol-4-yl)-2-(2-mercaptophenylamino)-6-(naphthalen-1-yl) nicotinonitrile (**14**). Yield 74%, brown powder, m.p. 108–110 °C; IR (KBr): ν (cm^−1^) 3330 (NH), 2208 (C≡N), ^1^H-NMR (DMSO-d_6_): δ (ppm) 9.29 (s, 1H, pyrazole-5H), 9.06–8.54 (m, 4H, Ar-H, thionyl-H), 8.26–7.66 (m, 7H, Ar-H for naphthalene), 7.60–6.66 (m, 10H, Ar-H), 3.34 (s, 1H, NH, disappeared in D2O), 1.20 (s, 1H, SH, disappeared in D_2_O). MS *m*/*z* (ESI): 589 [M^+^] (32), 465 (82), 441 (62), 237 (100), 127(12), 124 (20); Anal. Calcd. for C_37_H_24_FN_5_O (589.60): C, 75.36, H, 4.10; N, 11.88. Found C, 75.18; H, 4.05; N, 11.73%.

4-(3-(4-Fluorophenyl)-1-phenyl-1*H*-pyrazol-4-yl)-2-morpholino-6-(naphthalen-1-yl)nicotino- nitrile (**15a**). Yield 65%, pale brown powder, m.p. 130–133 °C; IR (KBr): ν (cm^−1^) 2226 (C≡N), ^1^H-NMR (DMSO-d_6_): δ (ppm) 9.16 (s, 1H, pyrazole-5H), 8.71–7.56 (m, 7H, Ar-H for naphthalene), 7.55–7.15 (m, 10H, Ar-H), 3.76 (t, 4H, *J* = 8.8 Hz), 3.05 (t, 4H, *J* = 8.8 Hz), MS *m*/*z* (ESI): 552 [M^+^] (52), 465 (28), 237 (100), 230 (7), 127 (12), 87 (22); Anal. Calcd. for C_35_H_26_FN_5_O (551.60): C, 76.21; H, 4.75; N, 12.70. Found C, 75.98; H, 4.26; N, 12.31%.

4-(3-(4-Fluorophenyl)-1-phenyl-1*H*-pyrazol-4-yl)-2-(4-methylpiperazin-1-yl)-6-(naphthalen-1-yl)nicotinonitrile (**15b**). Yield 61%, brown powder, m.p. 156–158 °C; IR (KBr): ν (cm^−1^) 2918 (aliph-H), 2227 (C≡N), ^1^H-NMR (DMSO-d_6_): δ (ppm) 9.18 (s, 1H, pyrazole-5H), 8.71–7.65 (m, 7H, Ar-H for naphthalene), 7.64–7.12 (m, 10H, Ar-H), 3.30–3.25 (m, 4H, 2CH_2_), 2.43–2.23 (m, 4H, 2CH_2_), 2.24 (s, 3H, CH_3_), MS *m*/*z* (ESI): 564 [M^+^] (27), 538 (25), 439 (12), 237 (100), 100 (23); Anal. Calcd. for C_35_H_29_FN_6_ (564.60): C, 76.58, H, 5.18; N, 14.88. Found C, 75.98; H, 4.92; N, 14.72%.

#### 4.1.8. Synthesis of 4-(3-(4-Fluorophenyl)-1-phenyl-1*H*-pyrazol-4-yl)-2-hydrazinyl-6-(naphthalen-1-yl)nicotinonitrile (**16**)

A mixture of the 2-chloronicotinonitrile **12** (5.0 g, 0.01 mol) and NH_2_NH_2_·H_2_O (0.04 mol) in EtOH (20 mL) was heated under reflux for 4h. The obtained solid was collected by filtration, dried and crystallized from EtOH/dioxane to yield **16**. Yield 86%, yellow powder, m.p. 164–168 °C; IR (KBr): ν (cm^−1^) 3417, 3310 (NH_2_), 3199 (NH), 2206 (C≡N), ^1^H-NMR (DMSO-d_6_): δ (ppm) 9.16 (s, 1H, pyrazole-5H), 8.35–7.97 (m, 7H, Ar-H for naphthalene), 7.96–6.88 (m, 10H, Ar-H), 4.82 (s, 1H, NH, disappeared in D_2_O), 3.43 (s, 2H, NH_2_, disappeared in D_2_O). ^13^C-NMR (DMSO-d_6_): δ (ppm) 149.3 (C-NHNH_2_), 148.3, 139.7 (C≡N), 139.2, 138.5, 136.1 (C=N), 135.3, 134.0, 133.8, 131.7, 131.5, 131.0, 130.9, 130.4, 130.2, 130.1, 129.5, 129.1, 128.1, 127.9, 127.6, 127.3, 126.9, 126.7, 126.4, 125.8, 125.4, 119.3 (C≡N), 118.2 (Ar-CH), 40.6, 40.0 (2CH); MS *m*/*z* (ESI): 496 [M^+^] (12), 465 (81), 440 (100), 237 (20), 204 (76); Anal. Calcd. for C_31_H_21_FN_6_ (496.55): C, 74.99; H, 4.26; N, 16.93. Found C, 74.86; H, 4.12; N, 16.78%.

#### 4.1.9. Synthesis of **17** and **18**

A mixture of **16** (4.9 g, 0.01 mol), acetylacetone or 4,4,4-trifluoro-1-(thiophen-2-yl)butane-1,3-dione (0.01 mol) in EtOH (10 mL) and AcOH (4 mL) was heated reflux for 3 h. After cooling, the solid obtained was filtered off, dried and crystallized from EtOH/dioxane to afford **17** and **18**, respectively.

2-(3,5-Dimethyl-1*H*-pyrazol-1-yl)-4-(3-(4-fluorophenyl)-1-phenyl-1*H*-pyrazol-4-yl)-6- (naphthalen-1-yl)nicotinonitrile (**17**). Yield 85%, pale orange powder, m.p. 270–272 °C; IR (KBr): ν (cm^−1^) 2209 (C≡N), 1620 (C=N), ^1^H-NMR (DMSO-d_6_): δ (ppm) 9.24 (s, 1H, pyrazole-5H), 8.17–7.96 (m, 7H, Ar-H for naphthalene), 7.66–7.35 (m, 10H, Ar-H), 7.25 (s, 1H, pyrazole-4H), 2.48 (s, 6H, 2 CH_3_); MS *m*/*z* (ESI): 560 [M^+^] (13), 533 (26), 438 (62), 237 (15), 95 (100); Anal. Calcd. for C_36_H_25_FN_6_ (560.60): C, 77.13; H, 4.49; N, 14.99. Found C, 76.92; H, 4.32; N, 14.81%.

4-(3-(4-Fluorophenyl)-1-phenyl-1*H*-pyrazol-4-yl)-6-(naphthalen-1-yl)-2-(5-(thiophen-2-yl)-3-(tri-fluoromethyl)-1*H*-pyrazol-1-yl)nicotinonitrile (**18**). Yield 82%, dark yellow powder, m.p. 117–119 °C; IR (KBr): ν (cm^−1^) 2209 (C≡N), ^1^H-NMR (DMSO-d_6_): δ (ppm) 8.92 (s, 1H, pyrazole-5H), 8.03–7.89 (m, 7H, Ar-H for naphthalene), 7.59–7.54 (m, 3H, thionyl-H), 7.53–7.33 (m, 10H, Ar-H), 6.88 (s, 1H, pyrazole-4H); MS *m*/*z* (ESI): 583 [M^+^] (10), 465 (72), 237 (100), 299 (8), 217 (5); Anal. Calcd. for C_39_H_22_F_4_N_6_S (682.60): C, 68.61; H, 3.25; N, 12.31. Found C, 68.02; H, 3.12; N, 12.03%.

#### 4.1.10. Synthesis of **19** and **20**

A solution of **16** (4.9 g, 0.01 mol) in a mixture of AcOH/Ac_2_O (10 mL) or in glacial AcOH (10 mL) was refluxed for 2 h, poured on ice/water, filtered off and crystallized from EtOH/dioxane to give **19** and **20**, respectively. Also, refluxing of **19** (0.5 g, 0.01 mol) in acetic anhydride (7 mL) afforded compound **20**.

4-(3-(4-Flurophenyl)-1-phenyl-1*H*-pyrazol-4-yl)-6-(naphthalen-1-yl)-1*H*-pyrazolo[3,4-b]pyridin-3-amine (**19**). Yield 84%, pale yellow powder, m.p. 140–143 °C; IR (KBr): ν (cm^−1^) 3425–3354 (NH_2_), 3198 (NH), ^1^H-NMR (DMSO-d_6_): δ (ppm) 8.92 (s, 1H, pyrazole-5H), 8.22–7.90 (m, 7H, Ar-H for naphthalene), 7.66–7.34 (m, 10H, Ar–H), 5.02 (s, 2H, NH2, disappeared in D_2_O), 4.63 (s, 1H, NH, disappeared in D_2_O); MS *m*/*z* (ESI): 496 [M^+^] (28), 479 (76), 244 (50), 237 (100); Anal. Calcd. for C_31_H_21_FN_6_ (496.52): C, 74.99; H, 4.26; N, 16.93. Found C, 74.76; H, 4.15; N, 16.82%.

*N*-(4-(3-(4-Flurophenyl)-1-phenyl-1*H*-pyrazol-4-yl)-6-(naphthalen-1-yl)-1*H*-pyrazolo-[3,4-b] pyridin-3-yl)acetamide (**20**). Yield 78%, yellow powder, m.p. 138–140 °C; IR (KBr): ν (cm^−1^) 3196 (NH), 1690 (C=O), ^1^H-NMR (DMSO-d_6_): δ (ppm) 12.37 & 10.31 (s, NH, OH), 8.88 (s, 1H, pyrazole- 5H), 7.98–7.59 (m, 7H, Ar-H for naphthalene), 7.57–6.88 (m, 10H, Ar-H), 4.82 (s, 1H, NH, disappeared in D_2_O), 2.73 (s, 3H, acetyl); MS *m*/*z* (ESI): 538 [M^+^] (20), 479 (36), 244 (20), 237 (100); Anal. Calcd. for C_33_H_23_FN_6_O (538.59): C, 73.59; H, 4.30; N, 15.60. Found C, 73.28; H, 4.19; N, 15.32%.

#### 4.1.11. Synthesis of *N*-(4-chlorobenzylidene)-4-(3-(4-fluorophenyl)-1-phenyl-1*H*-pyrazol-4-yl)-6- (naphthalen-1-yl)-1*H*-pyrazolo[3,4-b]pyridine-3-amine (**21**)

A solution of **16** or **19** (0.01 mol) in AcOH (10 mL) in the presence of 4-chlorobenzaldehyde (0.01 mol) was heated under reflux for 2 h, left to precipitate, filtered and crystallized from EtOH/ dioxane to afford **21**. Yield 58%, yellow powder, m.p. 158–160 °C; IR (KBr): ν (cm^−1^) 3192 (NH), ^1^H-NMR (DMSO-d_6_): δ (ppm) 9.89 (s, 1H, pyrazole-5H), 9.06 (s, 1H, N=C-H), 8.87–7.56 (m, 7H, Ar-H for naphthalene), 7.52–6.88 (m, 14H, Ar-H), 4.82 (s, 1H, NH, disappeared in D_2_O); MS *m*/*z* (ESI): 621 [M^+^] (15), 619 (48), 479 (20), 237 (80), 139 (35), 137 (100); Anal. Calcd. for C_38_H_24_ClFN_6_ (619.10): C, 73.72; H, 3.91; N, 13.57. Found C, 73.25; H, 3.82; N, 13.27%.

#### 4.1.12. Synthesis of 2-(4-(3-(4-fluorophenyl)-1-phenyl-1*H*-pyrazol-4-yl)-6-(naphthalen-1-yl)-1*H*- pyrazolo[3,4-b]-pyridin-3-yl)isoindoline-1,3-dione (**22**)

A mixture of **16** or **19** (0.01 mol) and tetrachlorophthalic anhydride (0.01 mol) in glacial acetic acid (10 mL) was refluxed for 1 h, poured on ice water, filtered off and crystallized from EtOH/dioxane to yield **22**. Yield 94%, yellow powder, m.p. 115–117 °C; IR (KBr): ν (cm^−1^) 3196 (NH), 1785, 1731 (C=O); ^1^H-NMR (DMSO-d_6_): δ (ppm) 8.87 (s, 1H, pyrazole-5H), 8.04–7.56 (m, 7H, Ar-H for naphthalene), 7.55–7.33 (m, 10H, Ar-H), 4.28 (s, 1H, NH, disappeared in D_2_O); Anal. Calcd. for C_39_H_19_Cl_4_FN_6_O_2_ (764.42): C, 61.28; H, 2.51; N, 10.99. Found C, 61.00; H, 2.42; N, 10.89%.

#### 4.2.13. Synthesis of 7-(3-(4-fluorophenyl)-1-phenyl-1*H*-pyrazol-4-yl)-5-(naphthalen-1-yl)-3-thioxo- 2,3-dihydro[1,2,4]triazolo[4,3-a]pyridine-8-carbonitrile (**23**)

Solution of hydrazinyl derivative **16** (4.9 g, 0.01 mol) in alcoholic KOH (10%, 20 mL) and CS_2_ (0.01 mol) was refluxed for 2 h, lift overnight, then poured on ice water, filtered off the solid obtained and crystallized from EtOH/dioxane to afford **23**. Yield 47% yellow powder, m.p. 288–290 °C; IR (KBr): ν (cm^−1^) 3192 (NH), 2218 (C≡N), 1240 (C=S); ^1^H-NMR (DMSO-d_6_): δ (ppm) 8.73 (s, 1H, pyrazole-5H), 7.97–7.63 (m, 7H, Ar-H for naphthalene), 7.53–6.77 (m, 10H, Ar-H), 3.76 (s, 1H, NH, disappeared in D_2_O). ^13^C-NMR (DMSO-d_6_): δ (ppm) 148.1 (C=S), 142.3, 138.7 (C=N), 133.8 (2), 133.4 (C=N), 131.7 (2), 131.2, 130.6 (2), 130.1, 129.9 (2), 129.4, 129.2 (2), 128.9, 128.4 (2), 126.9, 126.4 (2), 126.3 (2), 125.9 (2), 119.1 (C≡N), 110.0 (Ar-CH), 40.5, 39.9 (2CH); MS *m*/*z* (ESI): 538 [M^+^] (45), 494 (18), 479 (10), 453 (50), 237 (100); Anal. Calcd. for C_32_H_19_FN_6_S (538.60): C, 71.36; H, 3.56; N, 15.60. Found C, 71.31; H, 3.52; N, 15.58%.

### 4.2. Cytotoxicity Assay

#### 4.2.1. Materials and Cell Lines

Hepatocellular carcinoma (HepG2) and cervical Carcinoma (HeLa) cell lines, ATCC, VA, USA, were used throughout the work. All used chemicals and reagents were of high purity-cell culture grade.

#### 4.2.2. MTT Assay

Cytotoxic assay depends on the formation of purple formazan crystals by the action of dehydrogenase in living cells. Cells were cultured in RPMI-1640 medium supplemented with 10% fetal bovine serum, antibiotic solution (100 units/mL penicillin, 100 µg/mL streptomycin) at 37 °C in a 5% CO2 incubator. Cells were seeded in a 96-well plate (10^4^ cells/well), and the plates were incubated for 48 h. Afterwards, cells were exposed to variable concentrations of prepared derivatives and incubation proceeded for further 24 h. After treatment, 20 µL of MTT solution (5 mg/mL) was added and incubated for 4 h. DMSO (100 µL/well) is added and the developed color density was measured at 570 nm using a plate reader (ELx 800, BioTek, Winuski, VT, USA). Relative cell viability was calculated as (Atreated/Auntreated) ×100 [36,37]. Results were compared with doxorubicin as a positive control.

## 5. Conclusions

During the current investigation, we synthesized a new building block; namely 4-(3-(4-fluorophenyl)-1-phenyl-1*H*-pyrazol-4-yl)-2-hydroxy-6-(naphthalen-1-yl)nicotinonitril, with the help of multicomponent reaction systems. From that compound, a series of **16** different nicotinonitril derivatives were synthesized, and their structural and spectral data were elucidated. Furthermore, in vitro cytotoxic activities against hepatocellular and cervical carcinoma cell lines were investigated. Obtained results revealed that different synthesized compounds showed promising in vitro cytotoxic activities against both HepG2 and HeLa cell lines. Compounds **13** and **19** showed the most potent cytotoxic effect (IC_50_: 8.78 ± 0.7, 5.16 ± 0.4 μg/mL, and 15.32 ± 1.2 and 4.26 ± 0.3 μg/mL for HepG2 and HeLa cells, respectively.

## Figures and Tables

**Table 1:** IC_50_ values obtained for the tested compounds against both HepG2 and HeLa cell lines.

Compound	IC_50_ (µM) *	
	HepG2	HeLa
**8**	20.00 ± 1.7	35.58 ± 2.6
**9**	42.95 ± 3.2	55.00 ± 3.7
**10**	56.57 ± 3.4	47.02 ± 3.4
**11**	30.22 ± 2.1	43.64 ± 3.3
**12**	83.82 ± 4.5	89.72 ± 4.7
**13**	8.87 ± 0.70	15.32 ± 1.2
**14**	12.20 ± 1.0	19.44 ± 1.4
**15a**	90.05 ± 5.1	>100
**15b**	68.19 ± 3.7	75.05 ± 4.5
**16**	33.45 ± 2.3	10.37 ± 0.9
**17**	49.66 ± 3.2	65.91 ± 4.1
**18**	16.70 ± 1.3	7.67 ± 0.60
**19**	5.16 ± 0.40	4.26 ± 0.30
**20**	64.39 ± 3.6	28.15 ± 2.2
**21**	37.42 ± 2.5	24.83 ± 1.8
**22**	26.64 ± 1.9	9.33 ± 0.80
**23**	73.48 ± 4.0	62.07 ± 3.9
**Doxorubicin**	4.50 ± 0.20	5.57 ± 0.40

* IC_50_: 1–10 is (very strong), 11–20 is (strong), 21–50 is (moderate), 51–100 is (weak) and above is 100 (non-cytotoxic).

## References

[1] Rajeswari M., Saluja P., Khurana J.M. (2016). A facile and green approach for the synthesis of spiro[naphthalene-2,50-pyrimidine]-4-carbonitrile via a one-pot three-component condensation reaction using DBU as a catalyst. Rsc. Adv..

[2] El-Sayed H.A., Moustafa A.H., Haikal A.Z., Abu-El-Halawa R., El Ashry E.H. (2011). Synthesis, antitumor and antimicrobial activities of 4-(4-chlorophenyl)-3-cyano-2-(b-o-glycosyloxy)-6-(thien-2-yl)nicotine- nitrile. Eur. J. Med. Chem..

[3] Kotb E.R., El-Hashash M.A., Salama M.A., Kalf H.S., Abdel Wahed N.A.M. (2009). Synthesis and reactions of some novel nicotinonitrile derivatives for anticancer and antimicrobial evaluation. Acta Chim. Slov..

[4] Hamdy N.A., Anwar M.M., Abu-Zied K.M., Awad H.M. (2013). Synthesis, tumor inhibitory and antioxidant activity of new polyfunctionally 2-substituted 5,6,7,8-tetrahydronaphthalene derivatives containing pyridine, thioxopyridine and pyrazolopyridine moieties. Acta Polo. Pharm. Drug Res..

[5] Salem M.S., Sakr S.I., El-Senousy W.M., Madkour H.M.F., El-Senousy W.M. (2013). Synthesis, Antibacterial, and Antiviral Evaluation of New Heterocycles Containing the Pyridine Moiety. Arch. der Pharm..

[6] El-Sayed N.S., Shirazi A.N., El-Meligy M.G., El-Ziaty A.K., Rowley D., Sun J., Nagib Z.A., Parang K. (2014). Synthesis of 4-aryl-6-indolylpyridine-3-carbonitriles and evaluation of their anti-proliferative activity. Tetra. Lett..

[7] Ruiz J.F.M., Kedziora K., Keogh B., Maguire J., Reilly M., Windle H., Kelleher D.P., Gilmer J.F. (2011). A double prodrug system for colon targeting of benzenesulfonamide COX-2 inhibitors. Bioorganic Med. Chem. Lett..

[8] Balsamo A., Coletta I., Guglielmotti A., Landolfi C., Mancini F., Martinelli A., Milanese C., Minutolo F., Nencetti S., Orlandini E. (2003). Synthesis of heteroaromatic analogues of (2-aryl-1-cyclopentenyl-1-alkylidene)-(arylmethyloxy)amine COX-2 inhibitors: effects on the inhibitory activity of the replacement of the cyclopentene central core with pyrazole, thiophene or isoxazole ring. Eur. J. Med. Chem..

[9] Karrouchi K., Radi S., Ramli Y., Taoufik J., Mabkhot Y.N., Al-Aizari F.A., Al-Aizari F., Ansar M., A Al-Aizari F., Ansar M. (2018). Synthesis and Pharmacological Activities of Pyrazole Derivatives: A Review. Molecules.

[10] Bekhit A.A., Ashour H.M., Ghany Y.S.A., Bekhit A.E.-D.A., Baraka A. (2008). Synthesis and biological evaluation of some thiazolyl and thiadiazolyl derivatives of 1H-pyrazole as anti-inflammatory antimicrobial agents. Eur. J. Med. Chem..

[11] Christodoulou M.S., Liekens S., Kasiotis K.M., Haroutounian S.A. (2010). Novel pyrazole derivatives: Synthesis and evaluation of anti-angiogenic activity. Bioorganic Med. Chem..

[12] Bondock S., Fadaly W., Metwally M.A. (2010). Synthesis and antimicrobial activity of some new thiazole, thiophene and pyrazole derivatives containing benzothiazole moiety. Eur. J. Med. Chem..

[13] Chimenti F., Bolasco A., Manna F., Secci D., Chimenti P., Befani O., Turini P., Giovannini V., Mondovi B., Cirilli R. (2004). Synthesis and Selective Inhibitory Activity of 1-Acetyl-3,5-diphenyl-4,5-dihydro-(1H)-pyrazole Derivatives against Monoamine Oxidase. J. Med. Chem..

[14] Rashad A.E., Hegab M.I., Abdel-Megeid R.E., Micky J.A., Abdel-Megeid F.M. (2008). Synthesis and antiviral evaluation of some new pyrazole and fused pyrazolopyrimidine derivatives. Bioorganic Med. Chem..

[15] Bonesi M., Loizzo M.R., Statti G.A., Michel S., Tillequin F., Menichini F. (2010). The synthesis and Angiotensin Converting Enzyme (ACE) inhibitory activity of chalcones and their pyrazole derivatives. Bioorganic Med. Chem. Lett..

[16] Mahmoud M.R., El-Ziaty A.K., Abu El-Azm F.S.M., Ismail M.F., Shiba S.A. (2013). Utility of Cyano-N-(2-oxo-1,2-dihydroindol-3-ylidene)acetohydrazide in the Synthesis of Novel Heterocycles. J. Chem..

[17] El-Sayed N.S., Shirazi A.N., El-Meligy M.G., El-Ziaty A.K., Nagieb Z.A., Parang K., Tiwari R.K. (2016). Design, synthesis, and evaluation of chitosan conjugated GGRGDSK peptides as a cancer cell-targeting molecular transporter. Int. J. Boil. Macromol..

[18] El-Ziaty A.K., Shiba S.A. (2007). Antibacterial activities of new (E) 2-cyano-3-(3,4-dimethoxyphenyl) -2-propenoylamide derivatives. Synth. Commun..

[19] Mahmoud M.R., Shiba S.A., El-Ziaty A.K., Abu El-Azm F.S.M., Ismail M.F. (2014). Synthesis and reactions of novel 2,5-disubistituted 1,3,4-thiadiazoles. Synth. Commun..

[20] El-Shahawi M.M., El-Ziaty A.K. (2017). Enaminonitrile as Building Block in Heterocyclic Synthesis: Synthesis of Novel 4H -Furo[2,3-d ][1,3]oxazin-4-one and Furo[2,3- d ]pyrimidin-4 (3H) -one Derivatives. J. Chem..

[21] Mahmoud M.R., El-Ziaty A.K., Hussein A.M. (2013). Synthesis and Spectral Characterization of Novel Thiazolopyridine and Pyrimidine Derivatives. Synth. Commun..

[22] Ismail M.F., El-Sayed A.A. (2019). Synthesis and in-vitro antioxidant and antitumor evaluation of novel pyrazole-based heterocycles. Iran. Chem. Soc..

[23] Fahmy A.F.M., Rizk S.A., Hemdan M.M., El-Sayed A.A., Hassaballah A.I. (2018). Efficient Green Synthesis and Computational Chemical Study of Some Interesting Heterocyclic Derivatives as Insecticidal Agents. J. Chem..

[24] Rizk S.A., El-Sayed A.A., Mounier M.M., El-Sayed A.A. (2017). Synthesis of Novel Pyrazole Derivatives as Antineoplastic Agent. J. Chem..

[25] Fahmy A.F.M., El-Sayed A.A., Hemdan M.M. (2016). Multicomponent synthesis of 4-arylidene-2-phenyl-5(4H)-oxazolones (azlactones) using a mechanochemical approach. Chem. Central J..

[26] Hemdan M.M., El-Sayed A.A. (2016). Use of phthalimidoacetylisothiocyanate as a scaffold in synthesis of target heterocyclic systems with their antimicrobial assessment. Chem. Pharm. Bull..

[27] Hemdan M.M., El-Sayed A.A. (2016). Synthesis of some new heterocycles derived from novel 2-(1,3-dioxisoindolin-2-yl)benzoyl isothiocyanate. J. Heterocycl. Chem..

[28] Metwally M., Gouda M., Harmal A.N., Khalil A. (2012). Synthesis, antitumor, cytotoxic and antioxidant evaluation of some new pyrazolotriazines attached to antipyrine moiety. Eur. J. Med. Chem..

[29] Hossan A., Abu-Melha H. (2014). Synthesis, mass spectroscopic studies, cytotoxicity evaluation and quantitative structure activity relationship of novel isoindolin-1,3-dione derivatives. Chem. Process Eng. Res..

[30] Eissa I.H., El-Naggar A.M., El-Hashash M.A. (2016). Design, synthesis, molecular modeling and biological evaluation of novel 1H-pyrazolo[3,4-b]pyridine derivatives as potential anticancer agents. Bioorganic Chem..

[31] Shaaban S., Negm A., Sobh M.A., Wessjohann L.A. (2015). Organoselenocyanates and symmetrical diselenides redox modulators: Design, synthesis and biological evaluation. Eur. J. Med. Chem..

[32] Elsayed E.A., Farooq M., Dailin D., El-Enshasy H.A., Othman N.Z., Malek R., Danial E., Wadaan M. (2017). In vitro and in vivo biological screening of kefiran polysaccharide produced by Lactobacillus kefiranofaciens. Biomed. Res..

[33] Amr A.E.-G.E., El-Naggar M., Al-Omar M.A., Elsayed E.A., Abdalla M.M. (2018). In Vitro and In Vivo Anti-Breast Cancer Activities of Some Synthesized Pyrazolinyl-estran-17-one Candidates. Molecules.

[34] Amr A.E.-G.E., Abo-Ghalia M.H., Moustafa G.O., Al-Omar M.A., Nossier E.S., Elsayed E.A. (2018). Design, Synthesis and Docking Studies of Novel Macrocyclic Pentapeptides as Anticancer Multi-Targeted Kinase Inhibitors. Molecules.

[35] Dailin D.J., Elsayed E.A., Othman N.Z., Malek R., Phin H.S., Aziz R., Wadaan M., El Enshasy H.A. (2016). Bioprocess development for kefiran production by *Lactobacillus kefiranofaciens* in semi industrial scale bioreactor. Saudi J. Biol. Sci..

[36] Mosmann T. (1983). Rapid colorimetric assay for cellular growth and survival: Application to proliferation and cytotoxicity assays. J. Immunol. Methods.

[37] Denizot F., Lang R. (1986). Rapid colorimetric assay for cell growth and survival. Modifications to the tetrazolium dye procedure giving improved sensitivity and reliability. J. Immunol. Methods.

